# Task conflict in the Stroop task: When Stroop interference decreases as Stroop facilitation increases in a low task conflict context

**DOI:** 10.3389/fpsyg.2014.01182

**Published:** 2014-10-20

**Authors:** Benjamin A. Parris

**Affiliations:** Psychology Research Centre, Faculty of Science and Technology, Bournemouth UniversityPoole, UK

**Keywords:** Stroop, facilitation, interference, task conflict, goal

## Abstract

In the present study participants completed two blocks of the Stroop task, one in which the response-stimulus interval (RSI) was 3500 ms and one in which RSI was 200 ms. It was expected that, in line with previous research, the shorter RSI would induce a low Task Conflict context by increasing focus on the color identification goal in the Stroop task and lead to a novel finding of an increase in facilitation and simultaneous decrease in interference. Such a finding would be problematic for models of Stroop effects that predict these indices of performance should be affected in tandem. A crossover interaction is reported supporting these predictions. As predicted, the shorter RSI resulted in incongruent and congruent trial reaction times (RTs) decreasing relative to a static neutral baseline condition; hence interference decreased as facilitation increased. An explanatory model (expanding on the work of Goldfarb and Henik, 2007) is presented that: (1) Shows how under certain conditions the predictions from single mechanism models hold true (i.e., when Task conflict is held constant); (2) Shows how it is possible that interference can be affected by an experimental manipulation that leaves facilitation apparently untouched; and (3) Predicts that facilitation cannot be independently affected by an experimental manipulation.

## INTRODUCTION

The Stroop task requires participants to identify the color of the font in which a word is presented, whilst ignoring the meaning of the word itself. When the written word is incongruent with the font color (e.g., *green* written in brown), the time it takes to identify the color is increased relative to a baseline condition (i.e., a string of repeated letters, e.g., *xxxxx*, or a non-color related word, e.g., *stage*). The difference between the incongruent and baseline condition is known as *Stroop interference*. In contrast, when the color and word are congruent (e.g., *brown* written in brown) the time it takes to identify the color is decreased relative to the baseline condition; a difference referred to as *Stroop facilitation*. Stroop interference is putatively a consequence of response competition, and Stroop facilitation a consequence of response convergence, other mechanisms producing Stroop effects are thought to affect performance. In other words the two dimensions of the Stroop stimulus can provide evidence toward a response, resulting in competing or converging information.

### INFORMATIONAL CONFLICT AND CONVERGENCE AS DETERMINANTS OF STROOP TASK PERFORMANCE

Under most models of Stroop task performance, Stroop effects are the result of the same single mechanism. Under single mechanism accounts, information accruing from both dimensions of the Stroop stimulus comes together at the output or response module to either converge (facilitation) or compete (interference; see for example Cohen et al., 1990; Botvinick et al., 2001; Melara and Algom, 2003; Roelofs, 2003). For example, in the Cohen et al. (1990) model, and later implementations (e.g., Cohen and Huston, 1994; Botvinick et al., 2001), to ensure the response is based on color identity, a task demand unit biases activity in the color-processing pathway. This biasing also results in inhibition of the word processing pathway via recurrent inhibition (added in Cohen and Huston, 1994). If biasing is successful there is less information from the word dimension contributing converging and competing information, resulting in reduced facilitation and interference, respectively, by decreasing incongruent trial reaction times (RTs) and increasing congruent trial RTs. Such an account predicts that an experimental manipulation that influences how well the task demand unit biases activity should affect both indices of performance equally and in the same direction. In other words, this account and indeed intuitive conceptions of how interference and facilitation occur predict that a manipulation that affects one should affect the other in tandem (Brown, 2011) since a reduction in the contribution of the word dimension to the response level means less interference and less facilitation. However, a close look across studies employing the Stroop task reveals that whilst interference often affected by experimental manipulations facilitation appears to be unaffected (e.g., Tzelgov et al., 1992; MacLeod, 1998; Parris et al., 2012).

### TASK (SET) CONFLICT AS A DETERMINANT OF STROOP TASK PERFORMANCE

There is a large and growing literature on the role of task conflict in cognition (MacLeod and MacDonald, 2000; Monsell et al., 2001; Goldfarb and Henik, 2007; Steinhauser and Hübner, 2009; Kalanthroff and Henik, 2013; Braverman et al., 2014; see also Monsell, 2003 for a review of the task switching literature). Indeed MacLeod and MacDonald (2000) and Monsell et al. (2001) have argued that task set conflict contribute the Stroop effects. The argument is that there exists a more fundamental competition between the task sets of reading and color identification in the Stroop task that occurs independently of conflict that arises as a result of processing the informational content of each of the stimulus dimensions. When presented with a Stroop stimulus and endogenously preparing to respond to the color’s identity, an exogenously activated task set for word reading is activated that prepares cognitive machinery for decoding the visual symbols on the screen. As long as those symbols form a recognizable entry in the mental lexicon, the cognitive system activates a word reading task set. The simultaneous preparation of two task sets leads to task conflict even before information about the identity of both dimensions of the Stroop stimulus begins to compete or converge (MacLeod and MacDonald, 2000; Monsell et al., 2001; Steinhauser and Hübner, 2009; Braverman et al., 2014). Incongruent stimuli, non-color related neutral stimuli and congruent stimuli all consist of a word dimension and a color dimension and would therefore all be subject to this type of interference.

### DISSOCIATING INFORMATIONAL AND TASK LEVEL INFLUENCES

**FIGURE 1:**
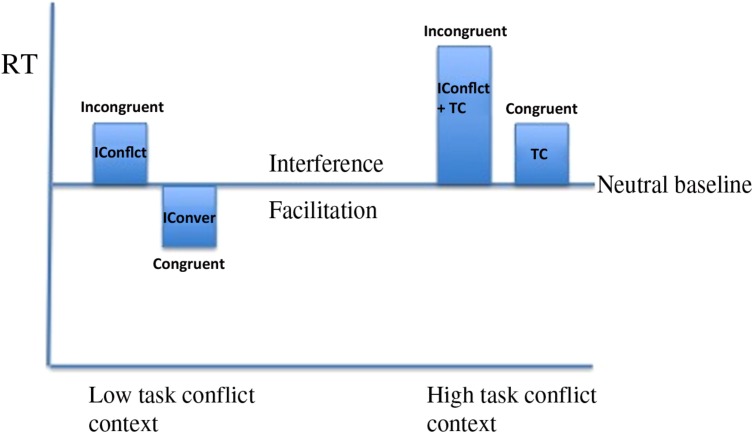
**Figure showing the effect of modifying the level of task conflict in the Stroop task.** In the low task conflict context RTs to incongruent trials and congruent trials are short revealing interference and facilitation effects due to Informational conflict (IConflict) and Informational Convergence (IConver), respectively. In the high task conflict context evidence for response facilitation disappears due to the overriding influence of task conflict (TC). However, from one context to another RTs to incongruent and congruent trials are affected in tandem whilst interference and facilitation effects are affected in opposing directions.

In an attempt to account for neural loci effects and to find support for the task conflict hypothesis, Goldfarb and Henik (2007; see also Kalanthroff et al., 2013) considered whether the behavioral expression of task conflict was being masked. Goldfarb and Henik (2007) distinguished between informational and task conflict and proposed the existence of a task conflict detector, which if operating quickly enough, could resolve task conflict leaving only informational competition or convergence to determine reaction times. In contrast, if task conflict is not resolved quickly it will be expressed in RTs potentially hiding informational contributions, leading to longer RTs for congruent than neutral trials; a reverse facilitation effect. Goldfarb and Henik (2007) employed a large proportion of repeated letter string (to be referred to as non-word neutral) trials to reduce the recruitment of the task conflict detector since such trials do not have a word dimension that could activate a word reading task set, and observed the predicted reverse facilitation effect. However, when a cue was presented that indicated an upcoming task conflict trial or an upcoming letter string trial, participants were able to prepare appropriate focus on the color identification goal, reducing RTs to congruent trials and eliminating the reverse facilitation effect. The reverse facilitation effect was interpreted as being due to increased task conflict in the absence of the cue. Importantly, in their study interference and facilitation were not affected in tandem but RTs to incongruent and congruent trials with both increasing in response to greater task conflict (although it is unclear whether the former reached significance). If both increase or decrease together whilst the baseline condition remains relatively unaffected it would modify the expression of interference and facilitation effects (see **Figure 1**).

Thus, the task conflict account predicts that both interference and facilitation could be affected independently and, importantly, in opposing directions. If an experimental manipulation enabled focusing on the color identification goal throughout the experiment, thereby reducing task conflict, and interference and facilitation were observable under such conditions, one should observe a reduction in interference (as a result of reduced task conflict) and a concurrent increase in facilitations effects (reduced task conflict leads to informational convergence being more apparent). Such evidence would be a challenge to single mechanism models and would provide further support for the role of task conflict in the Stroop and other conflict tasks. However, since Goldfarb and Henik (2007) observed no response time facilitation (neutral RTs > congruent RTs), their data cannot be marshaled as evidence for or against the notion that interference and facilitation are affected in tandem.

In the present study, an alternative manipulation to that employed by Goldfarb and Henik (2007) was used to modify attention to the color identification goal to permit the expression of informational convergence in the RT data. De Jong et al. (1999) used response-stimulus interval (RSI) to induce greater goal focus in a Stroop-like task. They reasoned that even assuming that participants were able to completely prevent word reading in the Stroop task, they might not always fully exploit this ability. In a typical Stroop experiment participants are likely to have to sit through numerous trials. It is likely that during the experiment participants will not be as focused as they could be on every trial. Indeed, De Jong et al. (1999) argued that it is not always strategically wise to employ fully focused attention on a task citing Simon (1994) who noted that a processing system that is fully absorbed in a task runs the risk of not noticing threatening events. De Jong et al. (1999) reasoned that a fast pace might induce or help participants to remain well focused on the instructed task. De Jong et al. (1999) showed that if RSI was shortened to 200 ms, the congruency effect (incongruent – congruent RTs) was substantially reduced and interpreted this result as evidencing greater goal focus in the short RSI condition and greater goal neglect in the longer RSI condition. Importantly, De Jong et al. (1999) did not include a neutral baseline condition in their study meaning that their data also cannot be marshaled as evidence for or against the notion that interference and facilitation effects are affected in tandem.

Thus, the aim of the present study was to use a short RSI manipulation to induce focus on the goal of color identification across an entire block of trials whilst including a neutral non-color word baseline. In the context of the dual-mechanisms of control theory (Braver, 2012) the shorter RSI is expected to increase pro-active control, although specifically task conflict control, not informational level control. The rationale for this was as follows: as noted above, previous work has argued for the presence of a form of conflict, beyond informational conflict, in the Stroop task (MacLeod and MacDonald, 2000; Monsell et al., 2001; Goldfarb and Henik, 2007). This conflict results from both word reading and color naming task sets being simultaneously activated. Goldfarb and Henik (2007) have shown that this competition can be heighten or lessened by increasing the proportion of trials without task conflict and trial type cueing, respectively, but that their particular manipulations eliminate Stroop facilitation effects (neutral trial RTs > congruent trial RTs). The RSI manipulation by De Jong et al. (1999) is similar to trial type cueing in that it lessens task conflict by having participants more strongly bias color identification. A major difference is that the RSI manipulation occurs across an entire block of trials meaning that participants do not have to suppress an automatically activated task set on a trial by trial basis but are permanently configured to ensure better control over task sets across the block.

It was expected that compared to the long RSI (3500 ms) condition the short RSI (200 ms) would increase focus on the color classification task, thereby reducing task conflict and decreasing interference, replicating the finding by De Jong et al. (1999). The low levels of task conflict were expected to permit the observation of facilitation effects in both RSI conditions. However, facilitation was predicted to be larger in the short RSI condition. This is because the present task conditions are effectively the opposite to the non-cued condition of Goldfarb and Henik (2007). In their study, they promoted task conflict so as to increase RTs to congruent trials, revealing, surprisingly, interference (congruent RTs > neutral RTs) on congruent trials. Here the aim was to use the short RSI to reduce task conflict so as to decrease RTs to congruent trials and, simultaneously, to incongruent trials.

Also in contrast to Goldfarb and Henik (2007) the present study employed non-color word neutral trials (e.g., stage) instead of an xxxx baseline condition. The exclusion of non word neutral trials (e.g., xxxx) ensured that the task conflict detector was in constant operation again engendering a low task conflict context ensuring Stroop facilitation effects could be observed in the RT data. Notably, since non-color word neutral trials also involve task conflict one might surmise that a reduction in task conflict induced by the shorter RSI would also lead to reduced RTs the neutral trials. However, MacLeod and MacDonald (2000) predicted that although non color-related neutral word stimuli would involve task conflict, they would likely lead to lower levels of task conflict than that observed with incongruent and congruent stimuli since incongruent and congruent stimuli are also part of the response set and thus their word representations would be more greatly activated throughout the experiment and thus greater task conflict. A reduction in task conflict would therefore be relatively more apparent on incongruent and congruent trials and RTs to neutral trials should be relatively less affected by the shorter RSI.

Whilst De Jong et al. (1999) did not distinguish between task and informational conflict, it was assumed that their interpretation of their results fits well with the notion that RSI acts to reduce task conflict. Indeed, in De Jong et al.’s (1999) study the RTs to both incongruent and congruent trials decreased in their short RSI condition (from 573 to 500 ms and from 526 to 489 ms, respectively) which is opposite to that predicted by single mechanism models under which RTs to incongruent trials would have decreased and RTs to congruent trials increased. Had there been a baseline control condition that was relatively unaffected by the RSI manipulation in their study they would have observed the decreased interference and increased facilitation predicted here (although had the neutral condition been similarly affected their result could not have been interpreted as clear evidence for or against a role for control processes). Therefore, if as was predicted, the short RSI leads to decreased interference and increased facilitation, such a finding would support the notion that the RSI manipulation operates over task conflict and that, whilst response competition and response convergence are important contributing factors to Stroop effects, extant single mechanism models would need to be modified to provide an important role for task conflict in determining Stroop effects. Indeed it would show it is not interference and facilitation but RTs to incongruent and congruent trials that are affected in tandem under present conditions.

## MATERIALS AND METHODS

### PARTICIPANTS

Thirty-five proficient English speakers from the student population at Bournemouth University participated. The average age of participants was 23.8 years (24 females). Participants received participation credits once they had completed the experiment. All participants signed a consent form prior to starting the experiment. No participants were excluded from the experiment.

### STIMULI AND MATERIALS

The font colors were brown, green, white, and yellow. The incongruent and congruent stimuli consisted of the color words *brown*, *yellow*, *green*, and *white* presented on a black background. The words used for the neutral stimuli were *stage*, *plenty*, *plane*, and *large* and were matched for frequency and word length using the MRC psycholinguistic database (http://websites.psychology.uwa.edu.au/school/MRCDatabase/uwa_mrc.htm). The stimuli were created in Microsoft Powerpoint using bold Courier New font, size 28. The visual angles subtended by the words were no smaller than 1.6∘ × 76∘ (17 mm long × 8 mm high) and no larger than 2.7∘ × 76∘ (28 mm long × 8 mm high). The stimuli were presented on a PC with a refresh rate of 60 Hz and a display resolution of 1920 × 1080. The experiment was programmed using Experiment Builder (SR Research). An Emprex chiclet keyboard was used to register responses.

### DESIGN

This experiment had a 2 (RSI: Short, Long) × 3 (Word Type: Incongruent, Neutral, Congruent) completely within-subjects design. The dependent variables were reaction time (in milliseconds) and percentage errors.

### PROCEDURE

Each participant completed 24 practice Stroop trials with an RSI of 2000 ms (a mid-way point between the two critical RSIs). No feedback was given to participants on their performance. Participants then completed 144 experimental trials in each RSI block consisting of 48 incongruent trials, 48 neutral trials and 48 congruent trials which were presented in random order. Each trial began with the presentation of a fixation cross for 200 or 3500 ms depending on RSI block, which was replaced with the Stroop stimulus presented until response. RSI block order was counterbalanced between participants. Participants were asked to respond as quickly and as accurately as possible to the color of the stimulus and ignore the meaning of the written word. Participants responded by pressing one of four color patches located on the z, x, n, and m keys on the keyboard (white, green, brown and yellow patches, respectively). Participants were offered the opportunity to take a 5 min break between blocks, but none accepted. The experiment took roughly 20 min for each participant to complete.

## RESULTS

Any reaction times >3 standard deviations from the mean were excluded from analysis as were trials with RTs < 250 ms. This resulted in 1.44% of the data being removed. 3.5% of the total number of trials were recorded as errors and were removed from the analysis of the reaction time data.

### ANALYSIS OF REACTION TIMES

**FIGURE 2:**
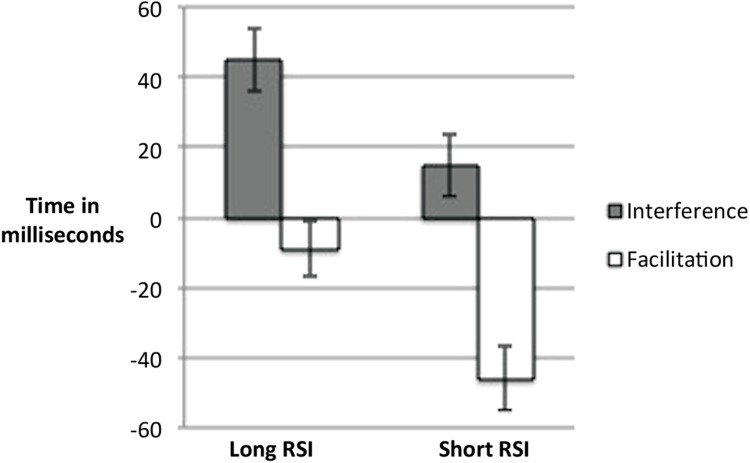
**Figure showing a crossover interaction where facilitation increases and interference decreases as RSI decreases**.

The data were entered into a 2 (RSI: Short, Long) × 3 (Word Type: Incongruent, Neutral, Congruent) repeated measures ANOVA. Analysis revealed a main effect of word type, *F*(2,68) = 31.050, *p* < 0.001, $\eta_{p}^{2}$ = 0.477, a main effect of RSI, *F*(1,34) = 6.063, *p* < 0.05, $\eta_{p}^{2}$ = 0.151, and an interaction between word type and RSI, *F*(2,68) = 6.638, *p* < 0.01, $\eta_{p}^{2}$ = 0.163. To investigate the interaction further, the data were non-orthogonally decomposed to look for partial interaction effects. Facilitation effects were investigated using a 2 (RSI: Short, Long) × 2 (Word Type: Congruent, Neutral) ANOVA which revealed main effect of word type, *F*(1,34) = 18.722, *p* < 0.001, $\eta_{p}^{2}$ = 0.335, a significant main effect of RSI, *F*(1,34) = 4.804, *p* < 0.05, $\eta_{p}^{2}$ = 0.124, and an interaction where *F*(1,34) = 11.594, *p* < 0.01, η^2^ = 0.254. This analysis showed that, as predicted, facilitation increased in the short RSI condition. The 2 (RSI: Short, Long) × 2 (Word Type: Incongruent, Neutral) ANOVA yielded a main effect of word type, *F*(1,34) = 21.161, *p* < 0.001, $\eta_{p}^{2}$ = 0.384, and no main effect of RSI *F*(1,34) = 3.186, *p* > 0.05. The interaction was significant where *F*(1,34) = 5.815, *p* < 0.05, $\eta_{p}^{2}$ = 0.146. This analysis showed that interference decreased in the short RSI condition, replicating the finding by De Jong et al. (1999). Overall this analysis revealed a crossover interaction. **Figure 2** depicts this interaction and **Table 1** shows the mean RTs and standard deviations as a function of RSI.

**Table 1:** Mean reaction times (milliseconds; standard deviations in brackets) magnitudes of interference (incongruent-neutral) and facilitation (neutral-congruent) and percentage errors as a function of response-stimulus interval.

	Response-stimulus interval	
	Long (3500 ms)	Short (200 ms)
Incongruent	790 (127) 3.2%	754 (97) 6%
Neutral	745 (124) 2.3%	739 (110) 3.3%
Congruent	737 (123) 2.8%	693 (101) 3.6%
***Interference***	45**	**15^∧^**
***Facilitation***	**8^∧^**	46**

*p < 0.001; ^∧^*p* > 0.1.

Paired-sample *t*-tests were carried out on the magnitudes of interference and facilitation in each condition. The results showed that interference was significant in the long RSI condition [45 ms; *t*(34) = 5.172, *p* < 0.001], but not in the short RSI condition [15 ms; *t*(34) = 1.655, *p* > 0.1]. In contrast, facilitation was not significant in the long RSI condition [8 ms; *t*(34) = 1.087, *p* > 0.2], but was significant in the short RSI condition [46 ms; *t*(34) = 5.214, *p* < 0.001]. Furthermore, comparison of RTs to neutral trials in the short RSI condition (Mean = 738.6 ms, SE = 18.6 ms) and neutral trials in the long RSI condition (Mean = 745.4 ms, SE = 18.6) revealed no significant difference where *t*(34) = 0.533, *p* > 0.5.

### ANALYSIS OF ERRORS

The 2 × 3 ANOVA analyzing the number of errors committed in each condition revealed a main effect of word type, *F*(1.938, 65.889) = 3.969 (Greenhouse–Geisser), *p* < 0.05, $\eta_{p}^{2}$ = 0.105, where there were a greater number of errors to incongruent trials (Mean = 2.086, SE = 0.243) than to congruent trials (Mean = 1.571, SE = 0.245) or neutral trials (Mean = 1.429, SE = 0.216) a main effect of RSI, *F*(1,34) = 22.076, *p* < 0.001, $\eta_{p}^{2}$ = 0.394, where there were more errors in the short RSI condition (Mean = 2.086, SE = 0.198) than in the long RSI condition (Mean = 1.305, SE = 0.205) but no interaction, *F*(1.993, 67.774) = 1.321 (Greenhouse–Geisser), *p* > 0.2. The main effect of RSI could be taken as evidence for the notion that the short RSI actually leads to increased task conflict (see **Table 1** for percentage errors by condition). This is fully discussed in the Discussion section below.

## DISCUSSION

The present study used a RSI manipulation to modify attention to competing task sets during Stroop task performance. In line with predictions, a crossover interaction was observed in which interference decreased as facilitation increased. Interestingly, the RTs to the neutral trials were not affected by the RSI manipulation. These results replicate the effect of the short RSI on interference observed by De Jong et al. (1999) and support a role for task conflict in determining Stroop interference (MacLeod and MacDonald, 2000; Monsell et al., 2001) and facilitation (Goldfarb and Henik, 2007). No reversed facilitation effects were observed in this study in the long RSI condition suggesting that the weaker goal focus did not lead to poor enough task conflict control to produce such an effect. It is likely that the exclusion of the non-word neutral baseline ensured that the task conflict detector was in constant operation throughout each block to prevent any such effects from being observed.

### IMPLICATIONS FOR SINGLE-MECHANISM MODELS

The observed results pose problems for single mechanism models that predict that Stroop interference and facilitation should be affected in tandem. Under these models, when focus to the color identification goal is increased, activation from the word dimension should decrease and resultantly decrease the availability of competing and converging information at the level of response output and with it interference and facilitation effects. Taken together with the work by Goldfarb and Henik (2007) and Kalanthroff et al. (2013), the present results suggest that single mechanism models will have to be modified. For example, the biased attention model of Cohen et al. (1990), Cohen and Huston (1994), and Botvinick et al. (2001) would need to incorporate a level where task set representations can compete and directly influence response output before competing or converging identity information from each dimension is sent to the response output level. Monsell et al. (2001) suggested that task nodes could have graded activation with a fixed quantity of activation divided between them. This would mean that the activation of one task node could be greater than the other, but that under certain contexts there would be equal activation of the task nodes, leading to greater task conflict. If sufficient biasing of attention from the color identification node were not received, connections to the response output level from the task set level would be more influential on responses than identity information.

### INCONGRUENT AND CONGRUENT RTs, NOT INTERFERENCE AND FACILITATION, ARE AFFECTED IN TANDEM

Whilst interference and facilitation were not affected in tandem, RTs to congruent and incongruent trials were. This effect is similar to one observed by Vanderhasselt et al. (2006) following rTMS over the left dorsolateral prefrontal cortex. Consistent with the argument put forward here Vanderhasselt et al. (2006) attributed the concomitant decrease to both trial types to the implementation of top-down attentional control. Had RSI only affected informational conflict and convergence a different pattern of results would have been observed. Importantly, and in contrast to De Jong et al. (1999) and Vanderhasselt et al. (2006), in the present study a neutral baseline condition was included, the RTs to which were not modified by the RSI manipulation. The static nature of RTs to this baseline condition means that as task conflict was reduced and RTs to incongruent and congruent trials both decreased, interference and facilitation decreased and increased, respectively (see **Figure 1**).

### ACCOUNTING FOR THE OPPOSING EFFECTS OF EXPERIMENTAL MANIPULATIONS ON INTERFERENCE AND FACILITATION

**FIGURE 3:**
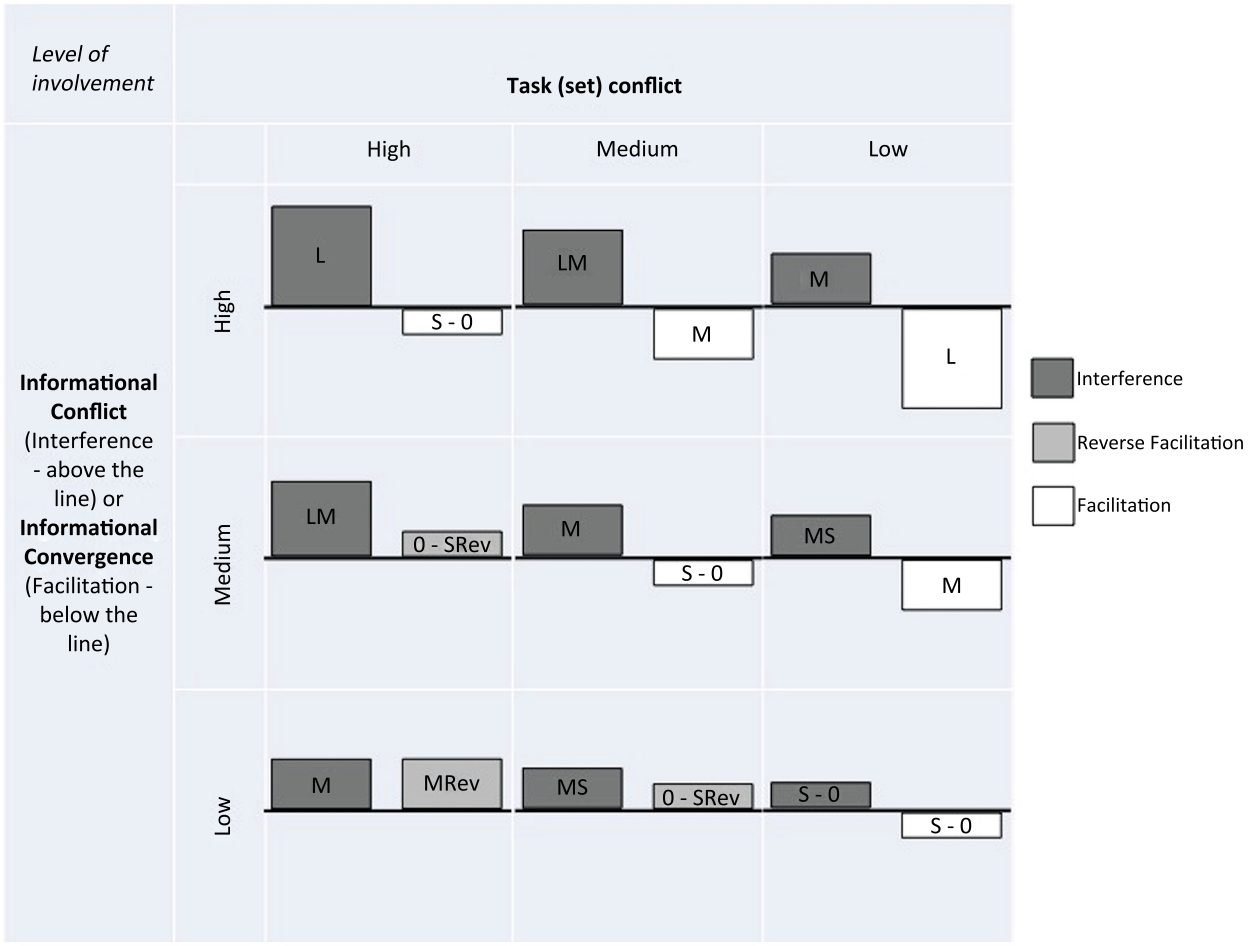
**Figure showing how Stroop interference, reverse facilitation and facilitation are modified by high, medium, and low levels of involvement of Informational- and Task-level mechanisms.** L, Large; M, Medium; S, Small; Rev, Reverse Facilitation. In this scheme the short RSI manipulation likely results in High to Medium Informational-level involvement and low Task-level involvement. The greater proportion of non-word neutral trials used by Goldfarb and Henik (2007) likely results in Medium to Low Informational-level involvement and High Task-level involvement. Their cueing manipulation likely reduced Task-level involvement to Medium.

The present results suggest that the task conflict account of Stroop effects provides a useful and powerful explanatory mechanism. However, the question remains as to whether it can account for the common observance of experimental effects influencing interference but not facilitation (Tzelgov et al., 1992; MacLeod, 1998; Parris et al., 2012) or *vice versa* in conditions such as Schizophrenia (Barch et al., 1999). **Figure 3** shows how different levels of involvement of both of these mechanisms could lead to varying patterns of interference, reverse facilitation and facilitation. An increase in informational and task conflict would both increase RTs to incongruent trials and, assuming a static baseline condition, increase interference. However, an increase in informational convergence and task conflict would have opposing effects on facilitation since the former would decrease RTs to congruent trials and the latter would increase them, effectively canceling each other out. Thus in principle at least, an increase in task and informational level involvement could produce an increase in interference without apparently affecting facilitation. Goldfarb and Henik (2007) created a high task conflict context by increasing the proportion of trials that did not involve task conflict so that when a trial that did involve task conflict was experienced, the participant was not able to deal well with the task level conflict. Should one also increase the number of congruent trials to increase informational-level involvement (whilst keeping the proportion of trials that do not involve task conflict higher, and the number of incongruent trials lower) one might expect to observe an increase in the influence of both informational and task mechanisms on performance and thus the effects described above.

Parris et al. (2012) showed that a post-hypnotic suggestion that words will look like gibberish or words of a foreign language given to high hypnotisable individuals resulted in a reduction in Stroop interference from 56 to 6 ms. Although it has been argued that the suggestion works by actually de-automatising word reading (Raz et al., 2002; Lifshitz et al., 2013). However, if word reading were deautomatised it is difficult to see how facilitation (informational convergence) could be unaffected. Other work also questions this deautomatising interpretation and instead indicates that the suggestion operates directly over response competition (informational conflict) after the words have been processed (Augustinova and Ferrand, 2012; Parris et al., 2013), hence leaving facilitation untouched. It is currently unclear as to whether informational conflict and convergence could be orthogonally manipulated, but the present model does not need this to be true to account for Parris et al.’s (2012) finding. Such an effect could be caused by a decrease in both informational-level and task-level conflict. This would substantially reduce interference and simultaneously decrease (via reduced informational convergence) and increase (via decreased task conflict) facilitation, leaving the latter apparently untouched. Thus, word reading could be relatively deautomatised under this account and still result in facilitation effects. It also allows for a scenario in which both informational and task level involvement is reduced to 0 following complete deautomatisation leaving no interference or facilitation as observed by Raz et al. (2002).

Assuming that informational and task set level influences were the only ones contributing to indices of Stroop task performance, one prediction that follows from this account is that it would not be possible to affect facilitation independently of interference since modifying the input of both task set level and informational level mechanisms would always affect interference. Neither could individual differences in the functioning of these two mechanisms or their impairment result in differences in facilitation effects alone. The opposing influences of task and informational mechanisms on congruent trial RTs means that they could appear unaffected by an experimental manipulation. In contrast to such predictions, Barch et al. (1999) have shown that patients with schizophrenia present with increased facilitation effects but apparently unaffected levels of interference. However, they account for the lack of an effect of schizophrenia on interference by noting the much higher level of errors in the patients than the controls. This means that it is likely that the incongruent trials on which the patients had most difficulty were counted as errors and thus did not contribute to their overall average incongruent trial RT belying the difficulty they experienced. Instead their results are best interpreted as showing that interference and facilitation effects simultaneously increased which can be explained by task level involvement being held constant whilst informational level involvement increased. In **Figure 3** this equates to moving from, e.g., Medium Informational-level involvement and Medium Task-level involvement to High Informational-level involvement and Medium Task-level involvement; in other words patients with schizophrenia have difficulty with controlling informational-level involvement but not Task (set)-level involvement. This is interesting because it also highlights how under certain conditions predictions from single mechanism models can be upheld (i.e., when task level involvement is held constant).

### ARE THERE CONTRASTING RESULTS IN THE ERROR DATA?

The main effect of RSI in the error data appears to be opposite to that predicted by the notion that the shorter RSI improves goal focus. However, the number of errors committed is not modified by Word Type, whilst the effect of interest in the RT data is driven by an interaction. Nevertheless, with more incongruent, neutral and congruent errors in the short RSI condition than in the long RSI condition, respectively (see **Table 1**) it is possible that the pattern of error data are responsible for the observed effects in the RT data. For example, more errors in the short RSI incongruent condition compared to the long RSI incongruent condition could result in removing the incongruent trials from the short RSI condition on which participants experienced most difficulty, thereby removing their effect on the average RT rendering the average incongruent trial RT longer in the long RSI condition, which was observed here. However, like the RT data, consideration of the mechanisms underpinning the error data can be undertaken from two perspectives: (1) Assuming errors are the result of greater informational level involvement (consistent with the notion that the short RSI leads to reduced task conflict); (2) Assuming errors are the result of greater task-level involvement (inconsistent with the notion that the short RSI leads to reduced task conflict). In the following paragraphs these two possibilities are considered marshaling RTs to both error and correct trials as evidence.

Considering errors as the result of informational level involvement, if error trials are those trials on which participants experience the most difficulty, as opposed to being hasty random errors, then the average RT for error incongruent trials should be greater than those for correct incongruent trials. Numerically (the low and different number of errors in each condition would mean that a statistical comparison of the error and correct RTs is likely to be unreliable so was not undertaken), the data are consistent with this interpretation in the short RSI condition: The error RTs for incongruent trials were numerically longer than the correct RTs in the Short RSI condition (770 ms vs. 754 ms, respectively), but not in the Long RSI condition (721 ms vs. 790 ms, respectively). Applying this account to congruent RTs and the greater number of errors would result in removing the congruent trials on which participants had most difficulty ignoring the meaning of the word; in other words, those trials on which there was the greatest amount of facilitation. This would mean that error RTs would be shorter than those to congruent trial RTs and this is support by the data: Error RTs to congruent trials were shorter than correct RTs in the Short RSI condition (652 ms vs. 693 ms, respectively) as well as in the Long RSI condition (664 ms vs. 737 ms, respectively). Thus, comparisons of error and correct RTs support the notion that the error trials in the short RSI condition are the trials on which participants were less able to prevent the word dimension from affecting their responses at an informational level. This explanation does however account for the observed reduction in incongruent trials RTs and thus one does not necessarily need to invoke reduced task conflict as an account of the observed effects on interference. However, the effect of RSI on incongruent trials replicates the effect observed by De Jong et al. (1999), and they reported no main effect of RSI on errors with 3.8% errors in *both* the short RSI and long RSI incongruent conditions and nonetheless report significantly reduced incongruent trial RTs, which means that whilst errors might have contributed to the reduced incongruent RTs in the present study, other mechanisms must contribute to this effect. Furthermore, the decreased congruent trial RTs observed in this study is consistent with increased informational level involvement (as has been the argument throughout) and is consistent with the low task conflict account.

The above only applies if one assumes that the errors are driven by informational mechanisms. If error trials are in fact the trials on which participants experienced the greatest task conflict you would be removing those incongruent trials with the greatest amount of task conflict, thereby again reducing the average correct incongruent trial RT (as observed), but also removing those congruent trials that if included would increase the average correct congruent trial RT, and thus produce the decreased correct congruent trial RTs observed in the short RSI condition of the present study. However, greater task conflict on error trials predicts that the error congruent RTs that are removed would be longer than the correct congruent RTs. As reported above, error congruent RTs were shorter than correct congruent RTs in the short RSI condition. Thus, the data are once again inconsistent with the notion that the short RSI leads to greater task conflict.

Finally, one would expect to see a change to neutral trial RTs if errors were responsible for the patterns observed in the RT data. That is, if the (roughly) one extra error on incongruent trials in the short RSI condition were responsible for the 36 ms drop in RT, and the extra 0.6 errors on congruent trials were responsible for the 44 ms drop in RTs for congruent trials, with a difference of 0.6 errors committed in the two neutral conditions (half that observed on incongruent trials), one might expect for the decrease in RTs to neutral trials to also be significant, whereas the actual decrease in RTs on neutral trials is more than five times smaller than the decrease observed on incongruent trials trials (36.2 ms vs. 6.74 ms), and more than six times smaller than that observed on congruent trials and was not significant.

Thus, taking the present results and those of De Jong et al. (1999) together indicates that something other than errors are responsible for the reduced incongruent trial RTs in the short RSI condition. Furthermore, examining the RTs in the error data supports the notion that the short RSI condition results in low task conflict (and increased informational level conflict). Finally, the unmodified neutral trial RTs in the present study is also not consistent with the notion that the error data are responsible for the RT data.

### WHAT IS THE EFFECT OF A SHORT RSI ON PERFORMANCE?

In the present study it has been argued that the short RSI increases goal focus; an argument consistent with that made by De Jong et al. (1999). However, it is likely that other mechanisms are affected by the RSI manipulation. For example, it is conceivable that factors such as response or word repetition (priming from episodic memory; see Schmidt and DeHouwer, 2011, for a review) could be influenced by the shorter RSI. That is, with a shorter RSI the episodic trace from trial N-1 is more likely to be influential on trial N than when RSI is longer. Nevertheless, re-running the analysis reported above with word and response repetition trials excluded returns a significant 2 × 3 interaction (*p* < 0.01). This result shows that word and response repetition are not responsible for the reported effect.

Recent work has shown that conflict adaptation effects (shorter RTs on trials following incongruent trials) are larger at shorter RSIs in a Stroop-like task (Egner et al., 2010). Such an effect could explain some of the present results. However, two points mitigate this possibility: (1) If the present analysis is re-run removing all the trials where trial N-1 is incongruent, the pattern of results observed is identical to when they are included (Interference Long RSI = 40 ms vs. Interference Short RSI = 17 ms; Facilitation Long RSI = 13 ms vs. Facilitation Short RSI = 47 ms), although this does not quite reach significance (*p* = 0.092); (2) Like the present study Egner et al. (2010) observed a decrease in RT to incongruent trials in the short RSI condition, an effect explained by greater conflict adaptation effects, but they also observed an increase in RTs to congruent trials, the opposite to that observed in the present study (De Jong et al., 1999). Whilst there were differences between our two studies the similarities might suggest that we would expect to observe similar effects of the short RSI on both trials types. However, Egner et al. (2010) employed an RSI of 500 ms for their short RSI condition, not the 200 ms RSI that the present study and De Jong et al. (1999) employed. Similarly in a recent study Parris et al. (2012) employed an RSI manipulation in the Stroop task but employed a shorter RSI of 500 ms and did not observe any of the effects reported here [although this was with a special population (high suggestible individuals) and under special circumstances (in the context of a hypnosis study)]. Taken together the evidence suggests that the critical effects on goal focus appear to require a short RSI of ∼200 ms. Moreover, Egner et al. (2010) included response repetitions when calculating the conflict adaptation effects in their study which could be responsible for the effects observed in their study, but do not account for the results in the present study.

Finally, the shorter RSI might increase effects of response contingencies. Recent work has shown effects of contingency on congruent trial RTs (Schmidt et al., 2007; Schmidt and Besner, 2008). The contingency effect refers to the implicitly learned relationship between the word and response which is used to predict specific responses to each word. This is often the case when congruent trials are included in an experiment since they often make up half the trials. Since there are fewer possible congruent trials than incongruent trials, the color words in the experiments are more often associated with their color counterparts. Response contingency decreases RTs to trials where the correct response is highly correlated to the word. The observation of reduced RTs to congruent trials in the present experiment could be explained if one assumes that a shorter RSI leads to greater effects of response contingency. However, greater response contingency in the short RSI condition would also lead to increased RTs to incongruent trials; the opposite to that observed in the present study.

## SUMMARY AND CONCLUSION

In sum, the crossover interaction observed in the present study is strong evidence for a role for task conflict in producing Stroop effects. The present finding of the simultaneous increase in facilitation and decrease in interference is problematic for models of Stroop effects that predict these indices of performance should be affected in tandem. Instead the present results show that incongruent and congruent RTs are affected in tandem. An explanatory model (expanding on the work of Goldfarb and Henik, 2007) is presented that: (1) Shows how under certain conditions the predictions from single mechanism models hold true (i.e., when Task conflict is held constant); (2) Shows how it is possible that interference can be affected by an experimental manipulation that leaves facilitation apparently untouched; and (3) Predicts that facilitation cannot be independently affected by an experimental manipulation.

## Conflict of Interest Statement

The author declares that the research was conducted in the absence of any commercial or financial relationships that could be construed as a potential conflict of interest.
